# The Impact of Fish and the Commercial Marine Harvest on the Ocean Iron Cycle

**DOI:** 10.1371/journal.pone.0107690

**Published:** 2014-09-24

**Authors:** Allison R. Moreno, Arlene L. M. Haffa

**Affiliations:** Division of Science and Environmental Policy, California State University, Monterey Bay, Seaside, California, United States of America; College of Charleston, United States of America

## Abstract

Although iron is the fourth most abundant element in the Earth's crust, bioavailable iron limits marine primary production in about one third of the ocean. This lack of iron availability has implications in climate change because the removal of carbon dioxide from the atmosphere by phytoplankton requires iron. Using literature values for global fish biomass estimates, and elemental composition data we estimate that fish biota store between 0.7–7×10^11^ g of iron. Additionally, the global fish population recycles through excretion between 0.4–1.5×10^12^ g of iron per year, which is of a similar magnitude as major recognized sources of iron (e.g. dust, sediments, ice sheet melting). In terms of biological impact this iron could be superior to dust inputs due to the distributed deposition and to the greater solubility of fecal pellets compared to inorganic minerals. To estimate a loss term due to anthropogenic activity the total commercial catch for 1950 to 2010 was obtained from the Food and Agriculture Organization of the United Nations. Marine catch data were separated by taxa. High and low end values for elemental composition were obtained for each taxonomic category from the literature and used to calculate iron per mass of total harvest over time. The marine commercial catch is estimated to have removed 1–6×10^9^ g of iron in 1950, the lowest values on record. There is an annual increase to 0.7–3×10^10^ g in 1996, which declines to 0.6–2×10^10^ g in 2010. While small compared to the total iron terms in the cycle, these could have compounding effects on distribution and concentration patterns globally over time. These storage, recycling, and export terms of biotic iron are not currently included in ocean iron mass balance calculations. These data suggest that fish and anthropogenic activity should be included in global oceanic iron cycles.

## Introduction

The oceans provide ecosystem services that may be at risk due to human activity [1]. One service is the sequestration of atmospheric CO_2_ through photosynthesis, which mitigates climate change. Photosynthesis requires a sufficient supply of iron for the electron transfer mechanisms to proceed; however, in much of the ocean the concentration of iron in the oceans limits primary production [2]–[4]. While iron is abundant on earth, iron concentrations are limited in natural waters by its low solubility [5].

A biological need for iron is not limited to photosynthetic organisms. It is also required for respiration and nitrogen fixation; thus, most marine organisms concentrate iron in their bodies and cells. Many marine animals including fish, whales, turtles, and sharks have long distance migration patterns to support their mating, feeding, and spawning [6] that could help to transport iron and other nutrients to high nutrient-low chlorophyll regions of the ocean. It is estimated that the historical killing of baleen whales has removed ∼650 tonnes of iron from the Southern Ocean [7]. There may be compounding effects beyond the immediate removal of the elements contained in heterotrophic organisms from an ecosystem because these species excrete nutrient-containing waste. The feces of the baleen whales was found to have iron concentrations 6 orders of magnitude greater than the surrounding sea water [7]. Some current ocean iron cycles include internal cycling by phytoplankton, but higher trophic levels are not included [4], [5], [8], [9]. The purpose of this study is to estimate the amount of iron stored and recycled by fish, and to determine the amount of iron removed from the ocean due to the commercial marine harvest.

## Results

Global estimates of fish total biomass have recently been reported as between 8.99×10^11^–2.05×10^12^ kg [10]. Using elemental analysis data for whole fish we estimate that the global marine fish population stores between 0.7–7×10^11^ g of iron. The fertilizer effect by these organisms is even greater. We estimate that annual iron excretion by living fish ranges from 0.4–1.5×10^12^ g. Iron recycling by marine mammals and other animals is not included because we found it to be insignificant in comparison to the magnitude of that by bony fishes over the time range in this study. As noted above, historical whaling could be significant over longer time scales, or in some regions of the ocean [7].

**Figure 1:**
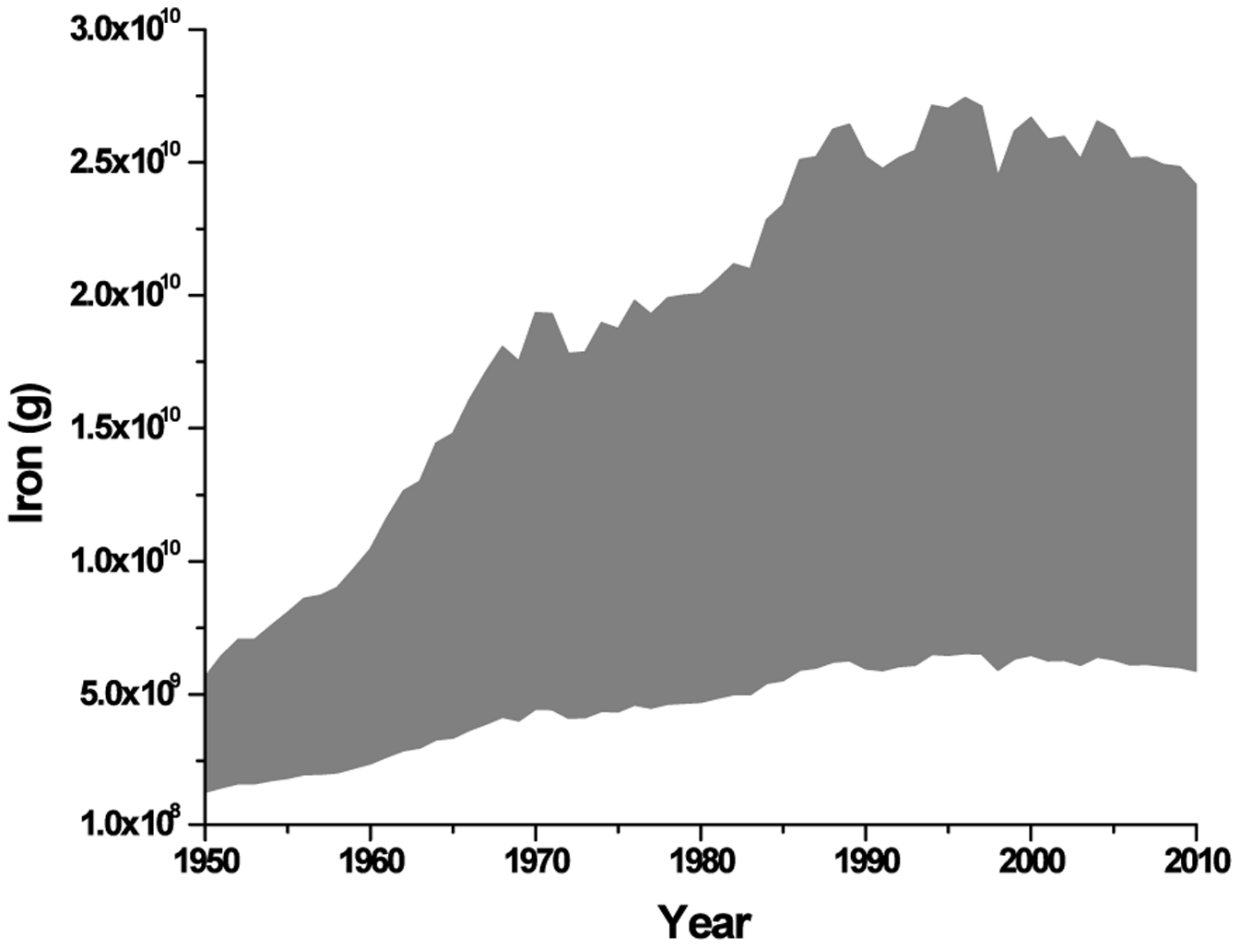
High and low estimates of iron removed from the ocean due to the commercial marine harvest. Range of estimates in gray from 1950 to 2010 (in grams per year). UN FAO [28] data obtained in tonnes (all non-mammal and 2 mammal species, n = 1567) or numbers (n = 83 mammal species) landed, and the highest and lowest elemental analysis data from the literature was used to estimate the range in grams removed. (See Table 1 and Database S1).

The translocation of iron from the ocean to land due to the commercial marine harvest, which includes species other than fish, is on the order of 1–30×10^9^ g per year (Database S1). Between 1950 and 2010 iron removal was lowest in 1950, reached a peak in 1996 and has declined since (Figure 1). While the marine harvest of iron is small on an annual basis, the export of stored iron integrated over this time range is ∼1×10^12^ g. The *Actinopterygii* (ray-finned fish) account for 62–82% of the total high and low estimates respectively (Table 1). Because the true catch data could be even higher our estimates are potentially more conservative than overstated. There have been concerns of under-reporting of fishing data to the UN for all countries except China [11]. Additionally, recreational fishing has estimates as high as 12% of the total commercial catch [12]–[14], and has increased over the past few decades [13], yet we do not take it into account due to lack of concrete data. The absolute loss term is compounded by the loss of nutrient cycling by these animals. The potential fecal fertilizer effect lost due to fishing is from 3–6×10^10^ g/yr.

**Table 1:** Summary of the iron content and average total annual iron removed.

Taxonomic Category	Representative Vernacular Nomenclature	Whole Organism Iron Content (g/kg)	Average Iron Removed per year (kg)
*Actinopterygi*	Ray-finned fishes	0.073–0.324 [29]	3,800,000–17,000,000
*Algae*	Seaweed, kelp, algae	0.050–0.737 [30]	1,600,000–2,000,000
*Chondrichthyes*	Sharks, skates, rays (cartilaginous fishes)	0.163 [31]	97,500
*Cnidaria*	Jellyfish, sea anemones, corals, hydra	0.053–0.088 [32]	7,400–12,000
*Crustacea*	Crab, shrimp, lobster, prawns	0.033–0.139 [29]	110,000–470,000
*Echinodermata*	Sea urchins, sea cucumbers, starfish	0.020–0.046 [32]	1,400–3,300
*Invertebrate*	Invertebrates	0.353–0.415 [30]	12,000–14,000
*Mammalia*	Dolphins, porpoises, seals, whales	0.0123–0.229 [33]	5,600–104,000
*Mollusca*	Sea snails, mussels, clams, squid, oysters, octopi, scallops	0.121–0.245 [29]	470,000–950,000
*Monocot*	Eel grass	0.022 [34]	15
*Myxini*	Hagfish, lampreys	0.034 [35]	25
*Nemertea*	Sea Worms	0.020–0.046 [29]	16–37
*Porifera*	Sponges	1.53 [36]	1,000
*Reptilia*	Turtles	0.522 [37]	1,500
*Tunicata*	Salps, sea squirts	0.18–0.500 [38]	690–1,900

Taxonomic categories used to calculate the marine harvest and the elemental iron composition. Average high and low estimates were obtained across the 61 year timespan from 1950 to 2010.

## Discussion

The chemistry of iron in the ocean is complicated and a detailed analysis of the global ocean iron cycle is beyond the scope of this paper. However, in order to assess the relative magnitude of iron stored in fish biomass and recycled in feces, as well as the marine harvest data it must be compared to estimates for bioavailable iron sinks and sources in the ocean. Currently recognized input, export, and assimilated storage terms include Aeolian dust, ice sheet melting, sediment deposition and upwelling, and plankton assimilation [4], [9], [15]–[18]. Bioavailable iron has been calculated as the total aqueous iron and between 1–10% of the colloidal and nanoparticulate iron. Using this calculation Aeolian dust inputs 0.6–2 Tg yr^−1^, iceberg melting inputs 0.09–0.1 Tg yr^−1^, and rivers input 0.08–0.09 Tg yr^−1^. [15] Recently a report that ice sheet melting provides 0.46–2.71 Tg yr^−1^ of nanoparticulate iron, of which 1–10% would be considered bioavailable using this calculation. An estimate for loss of iron from the cycle due to particulate iron sinking in the deep sea is 16 +/− 8 Tg yr^−1^ [19]. Particulate iron has very low solubility (<1–2%) which would limit its bioavailability [5]. The assimilation, and potential recycling into higher trophic levels by phytoplankton is estimated at 0.02–0.7 Tg yr^−1^. The phytoplankton associated iron and the iron stored in fish (0.07–0.7 Tg) and excreted by them (0.4–1.5 Tg yr^−1^) is 1.5–2.4 times more bioavailable than Fe_II_, the most bioavailable inorganic form [20]. Furthermore, the solubility and residence time of iron that is complexed with organic ligands is enhanced [21]. Iron from inorganic sources also has low solubility (≤2%) [5]; thus, transport and fertilization by fish could be a primary driver of marine primary productivity, especially in the high nutrient-low chlorophyll regions of the world. Fecal recycling of iron is roughly the same magnitude as the currently accepted greatest inputs due to sediment recycling and Aeolian dust. The annual loss of iron due to the marine harvest is only about 1% of the total available from inorganic inputs (0.006–.02 Tg yr^−1^); however, the cumulative removal of iron between 1950–2010 is 1.2×10^12^ g. A global median reduction of phytoplankton primary production at a similar rate of 1% annually has been observed over the past century. [22] The authors hypothesized that the reduced primary production was due to increases in sea surface temperature. This is supported by comprehensive studies of the effects of temperature on phytoplankton growth, but there is large variation between and within species. [23] Given the thermocline within the photic zone and the cooler temperatures within it, the increases in sea surface temperature may not account completely for this reduction in primary production, and it may be useful to include loss of iron due to fishing in future models. The removal of iron due to the commercial marine harvest is either essentially permanent on biological time scales or already included with fluvial or coastal input data as offal or wastewater. The majority of the fish are captured in upwelling zones and coastal areas. However, as fisheries have collapsed the industry has greatly expanded into off-shore [24] and deeper waters [25]. Thus if a portion of the iron removed via fishing is returned via sewage in riverine inputs, then this may represent a redistribution of iron even if the removal is mitigated by near shore replacement processes.

Most of the iron removed from the ocean is in the form of a complex higher life form at the top of food webs [14]. If iron-enrichment to reduce atmospheric CO_2_ is effected, or if global climate change causes physical processes to enhance the cycling of iron in regions of the ocean that are currently limited [4] the iron input will result in increases in primary production, or simple life forms at the bottom of food webs. How long the ocean will take to change from this trophic unsettling is not clear, however, if the longevity of many of the fish being removed is indicative, then it is on the order of decades. We conclude that the addition of marine animals as a biological reservoir and resource of organically-complexed iron, and marine harvest activities to ocean iron cycle models is warranted, and may ultimately lead to a better understanding of changes in biogeochemical processes over time.

## Materials and Methods

### Estimates for the amount of iron stored in fish tissue as a biological reservoir and recycled in the ocean due to feces

Global estimates of fish abundance have recently been reported as between 8.99×10^11^–2.05×10^12^ kg [10]. Elemental analysis data for whole fish was obtained from the literature and used to estimate the amount of iron stored in the global marine fish population (See literature sources in Table 1). Food consumption (Q) by body weight (B) of fish on an annual basis was obtained for all of the species in our list for which it was available (n = 54, Database S1) [26]. The average Q/B was 7.3, which is identical to a previously published estimate of 2% intake per body weight per day [27]. This was multiplied by global estimates of fish biomass [10] and then by the assimilation efficiency of iron in fish (0.7–1.2%, [27]). To obtain the most liberal range the low end biomass for both living and harvested fish was multiplied by the highest assimilation efficiency, and the greater biomass estimate with the lowest assimilation efficiency. Next we used these same estimates, but the loss term instead of the assimilation term to consider the fertilizer effect by these organisms. We estimate that annual iron excretion outputs of living fish range from 0.4–1.5×10^12^ g.

### Estimates of the loss term of iron due to the commercial marine harvest

The total commercial marine catch data for 1950 to 2010 was obtained from the Food and Agriculture Organization (FAO) of the United Nations, using FishStat software [22]. Freshwater organisms were excluded, and the data were then separated into the taxonomic categories: *Actinopterygii, Algae, Chondrichthyes, Cnidaria, Crustacea, Echinodermata, Invertebrate, Mammalia, Mollusca, Monocot, Myxini, Nemertea, Porifera, Reptilia*, and *Tunicata* (Table 1, Database S1). The database includes 1567 marine species in which the data were reported as mass landed, or brought to shore. For 83 species of mammals the data were reported as quantity caught, not tonnage. For these species high and low end estimates of male and female adult body weight [6] was used to obtain an estimate of the tonnage. High and low end values for elemental composition were obtained for each taxonomic category from the literature, and used to determine estimates of iron removal due to harvesting of that category (Summary in Table 1, full calculated dataset at Database S1). If the iron content was reported as a percentage of dry matter, then moisture content was used to determine percent iron for wet weights. The highest and lowest estimates for the fish in our database were also used to estimate the stored iron range in the living fish. Applying the same excretion calculations to the bony fish in this database we estimated the amount of recycled iron lost due to commercial fishing activities.

## Supporting Information

Database S1 **Marine Harvest Calculations and Food Consumption by Teleosts.** Commercial marine harvest data from 1950–2010 in tonnes for 1567 non-mammal species (tab 1), low Fe elemental composition for these species and an estimated low end value of Fe in this biomass (tab 2), high Fe elemental composition for these species and an estimated high end value of Fe in this biomass (tab 3), raw data of commercially harvested mammals 1950–2010 (tab 4), mammal harvest data in tonnes for 2 species and in number of animals caught for 83 species, and high and low estimates of Fe in their biomass (tab 5), food consumption data for 54 teleost species (tab 6). Source of raw data was the FAO Fisheries and Aquaculture of the United Nations [28]. Elemental analysis data sources provided in Table 1. Body mass estimates for mammalian species may be found in reference [6] and food consumption of teleosts in [26].
(XLS)

## References

[1] Worm B, Barbier EB, Beaumont N, Duffy JE, Folke C, et al (2006) Impacts of biodiversity loss on ocean ecosystem services. Science 314: 787–790 Available: http://www.sciencemag.org/content/314/5800/787.abstract. Accessed 2013 February 27 17082450 10.1126/science.1132294

[2] Hart T (1934) On the phytoplankton of the southwest Atlantic and the Bellingshausen Sea 1921–31. Discov Reports VIII: 1–268.

[3] Martin J, Fitzwater S (1988) Iron deficiency limits phytoplankton growth in the north-east Pacific subartic. Nature 331: 341–343.

[4] Misumi K, Lindsay K, Moore JK, Doney SC, Bryan FO, et al (2014) The iron budget in ocean surface waters in the 20th and 21st centuries: projections by the Community Earth System Model version 1. Biogeosciences 11: 33–55 Available: http://www.biogeosciences.net/11/33/2014/bg-11-33-2014.html. Accessed 2014 January 8

[5] Jickells TD, An ZS, Andersen KK, Baker AR, Bergametti G, et al (2005) Global iron connections between desert dust, ocean biogeochemistry, and climate. Science 308: 67–71. 15802595 10.1126/science.1105959

[6] Perrin, William F, Würsig B, Thewissen JGM, editors (2009) Encyclopedia of Marine Mammals. Second Edition. Amsterdam: Elsevier. 1414 p.

[7] Nicol S, Bowie A, Jarman S, Lannuzel D, Meiners KM, et al. (2010) Southern Ocean iron fertilization by baleen whales and Antarctic krill. Fish Fish 11: 203–209. Available: http://doi.wiley.com/10.1111/j.1467-2979.2010.00356.x Accessed 2013 March 5.

[8] Boyd PW, Ellwood MJ (2010) The biogeochemical cycle of iron in the ocean. Nat Geosci 3: 675–682. Available: http://www.nature.com/doifinder/10.1038/ngeo964 Accessed 2012 November 1.

[9] Fung I, Meyn S, Tegen I, Doney S, John J, et al (2000) Iron supply and demand in the upper ocean. Global Biogeochem Cycles 14: 281–295.

[10] Wilson RW, Millero FJ, Taylor JR, Walsh PJ, Christensen V, et al (2009) Contribution of fish to the marine inorganic carbon cycle. Science 323: 359–362. 19150840 10.1126/science.1157972

[11] Watson R, Pauly D (2001) Systematic distortions in world fisheries catch trends. Nature 414: 534–536. 11734851 10.1038/35107050

[12] Cooke SJ, Cowx IG (2004) The Role of Recreational Fishing in Global Fish Crises. Bioscience 54: 857–859. Available: 10.1641/0006-3568(2004)054[0857:TRORFI]2.0.CO;2

[13] Ihde TF, Wilberg MJ, Loewensteiner DA, Secor DH, Miller TJ (2011) The increasing importance of marine recreational fishing in the US: Challenges for management. Fish Res 108: 268–276. Available: 10.1016/j.fishres.2010.12.016 Accessed 2013 February 20.

[14] Pauly D, Christensen V, Guénette S, Pitcher TJ, Sumaila UR, et al (2002) Towards sustainability in world fisheries. Nature 418: 689–695. 12167876 10.1038/nature01017

[15] Raiswell R, Canfield D (2011) The Iron Biogeochemical Cycle Past and Present, Section 7 Iron sources to the oceans. Geochemical Perspect 1: 81–93.

[16] Moore JK, Doney SC, Lindsay K (2004) Upper ocean ecosystem dynamics and iron cycling in a global three-dimensional model. Global Biogeochem Cycles Available: https://darchive.mblwhoilibrary.org/handle/1912/3396. Accessed 2013 January 7.

[17] Moore JK, Braucher O (2008) Sedimentary and mineral dust sources of dissolved iron to the world ocean. Biogeosciences 5: 631–656.

[18] Hawkings JR, Wadham JL, Tranter M, Raiswell R, Benning LG, et al (2014) Ice sheets as a significant source of highly reactive nanoparticulate iron to the oceans. Nat Commun 5: 3929 Available: http://www.pubmedcentral.nih.gov/articlerender.fcgi?artid=4050262&tool=pmcentrez&rendertype=abstract. Accessed 2014 July 8 24845560 10.1038/ncomms4929 PMC4050262

[19] Raiswell R (2006) Towards a global highly reactive iron cycle. J Geochemical Explor 88: 436–439. Available: 10.1016/j.gexplo.2005.08.098 Accessed 2013 February 27.

[20] Andersen F, Lorentzen M, Waagbø R, Fisheries D (1997) Bioavailability and interactions with other micronutrients of three dietary iron sources in Atlantic salmon, *Salmo salar*, smolts. Aquacult Nutr 3: 239–246.

[21] Tagliabue A, Völker C (2011) Towards accounting for dissolved iron speciation in global ocean models. Biogeosciences 8: 3025–3039 Available: http://www.biogeosciences.net/8/3025/2011/. Accessed 2014 January 8

[22] Boyce DG, Lewis, Marlon R, Worm B (2010) Global phytoplankton decline over the past century. Nature: 591–596. 10.1038/nature09268 20671703

[23] Boyd PW, Rynearson TA, Armstrong EA, Fu F, Hayashi K, et al (2013) Marine phytoplankton temperature versus growth responses from polar to tropical waters–outcome of a scientific community-wide study. PLoS One 8: e63091 Available: http://www.pubmedcentral.nih.gov/articlerender.fcgi?artid=3660375&tool=pmcentrez&rendertype=abstract. Accessed 2014 July 23 23704890 10.1371/journal.pone.0063091 PMC3660375

[24] Swartz W, Sala E, Tracey S, Watson R, Pauly D (2010) The spatial expansion and ecological footprint of fisheries (1950 to present). PLoS One 5: e15143 Available: http://www.pubmedcentral.nih.gov/articlerender.fcgi?artid=2996307&tool=pmcentrez&rendertype=abstract Accessed 2014 July 15 21151994 10.1371/journal.pone.0015143 PMC2996307

[25] Norse EA, Brooke S, Cheung WWL, Clark MR, Ekeland I, et al (2012) Sustainability of deep-sea fisheries. Mar Policy 36: 307–320 Available: http://linkinghub.elsevier.com/retrieve/pii/S0308597X11001102. Accessed 2014 July 24

[26] Froese R, Pauly D, Editors (2014) Fishbase. World Wide Web electronic publication. Available: http://fishbase.org, version (06/2014).

[27] Bury N, Grosell M (2003) Iron acquisition by teleost fish. Comp Biochem Physiol Part C 135: 97–105 10.1016/S1532-0456 12860048

[28] FAO Fisheries & Aquaculture of the United Nations. FIGIS. - FishStat Plus - Universal software for fishery statistical time series (2012). Available: http://www.fao.org/fishery/statistics/software/fishstat/en.

[29] Bernard JB, Allen ME, et al (2002) Nutrition Advisory Group Handbook. Fact Sheet 005. Feeding Captive Piscivorous Animals: Nutritional Aspects of Fish as Food. Available: http://nagonline.net/810/feeding-captive-piscivorous-animals-nutritional-aspects-fish-food/Accessed 2012 June 8.

[30] Garcia-Casal MN, Pereira AC, Leets I, Ramirez J, Quiroga MF (2007) High Iron Content and Bioavailability in Humans from Four Species of Marine Algae. J Nutr 137: 2691–2695 Available: http://jn.nutrition.org/content/137/12/2691.long. Accessed 2013 February 6 18029485 10.1093/jn/137.12.2691

[31] Vannuccini S (1999) Shark Utilization, Marketing and Trade. Available: http://www.fao.org/docrep/005/x3690e/x3690e00.htm. Accessed 2014 April 9.

[32] Shiber JG (1981) Metal concentrations in certain coastal organisms from Beirut. Hydrobiologia 83: 181–195 Available: http://link.springer.com/10.1007/BF00008266 Accessed 2013 February 6

[33] Honda K, Yamamoto Y, Tatsukawa R (1987) Distribution of heavy metals in Antarctic marine ecosystem. Proc NIPR Symp Polar Biol: 184–197. Available: http://polaris.nipr.ac.jp/~penguin/polarbiosci/issues/pdf/1987-Honda.pdf. Accessed 2013 July 9.

[34] Holmer M, Pedersen O, Ikejima K (2006) Sulfur cycling and sulfide intrusion in mixed Southeast Asian tropical seagrass meadows. Bot Mar 49: 91–102 Available: http://www.degruyter.com/view/j/botm.2006.49.issue-2/bot.2006.013/bot.2006.013.xml Accessed 2013 May 23

[35] Chiu K-H, Mok H-K (2011) Study on the accumulation of heavy metals in shallow-water and deep-sea hagfishes. Arch Environ Contam Toxicol 60: 643–653 Available: http://www.ncbi.nlm.nih.gov/pubmed/20665212 Accessed 2013 June 6 20665212 10.1007/s00244-010-9572-8

[36] Padovan A, Munksgaard N, Alvarez B, McGuinness K, Parry D, et al (2012) Trace metal concentrations in the tropical sponge Spheciospongia vagabunda at a sewage outfall: synchrotron X-ray imaging reveals the micron-scale distribution of accumulated metals. Hydrobiologia 687: 275–288 Available: http://link.springer.com/10.1007/s10750-011-0916-9. Accessed 2013 July 12

[37] Kienzle E, Kopsch G, Koelle P, Clauss M (2006) The WALTHAM International Nutritional Sciences Symposia Chemical Composition of Turtles and Tortoises. 1–3: 2053–2054. 10.1093/jn/136.7.2053S 16772495

[38] Strohal P, Tuta J, Kolar Z, Limnology S, Mar N (1969) Investigations of Certain Microconstituents of Two Tunicates. Limnol Oceanogr 14: 265–268.

